# Association Between e-Cigarette Use and Depression in the Behavioral Risk Factor Surveillance System, 2016-2017

**DOI:** 10.1001/jamanetworkopen.2019.16800

**Published:** 2019-12-04

**Authors:** Olufunmilayo H. Obisesan, Mohammadhassan Mirbolouk, Albert D. Osei, Olusola A. Orimoloye, S. M. Iftekhar Uddin, Omar Dzaye, Omar El Shahawy, Mahmoud Al Rifai, Aruni Bhatnagar, Andrew Stokes, Emelia J. Benjamin, Andrew P. DeFilippis, Michael J. Blaha

**Affiliations:** 1 The American Heart Association Tobacco Regulation and Addiction Center, Dallas, Texas; 2 Johns Hopkins Ciccarone Center for the Prevention of Cardiovascular Disease, Baltimore, Maryland; 3 Department of Medicine, Yale New Haven Hospital, New Haven, Connecticut; 4 Department of Medicine, Vanderbilt University Medical Center, Nashville, Tennessee; 5 Johns Hopkins University, Baltimore, Maryland; 6 Department of Radiology and Neuroradiology, Charité, Berlin, Germany; 7 Section on Tobacco, Alcohol, and Drug Use, Department of Population Health, School of Medicine, New York University, New York; 8 Public Health Research Center, New York University, Abu Dhabi, United Arab Emirates; 9 Section of Cardiology, Department of Medicine, Baylor College of Medicine, Houston, Texas; 10 University of Louisville, Louisville, Kentucky; 11 Boston University, Boston, Massachusetts

## Abstract

**Importance:**

The prevalence of the use of electronic cigarettes (e-cigarettes) in the United States has grown rapidly since their introduction to the market more than a decade ago. While several studies have demonstrated an association between combustible cigarette smoking and depression, the association between e-cigarette use and depression has not been thoroughly studied.

**Objective:**

To examine the association between e-cigarette use and depression in a nationally representative sample of the adult population in the United States.

**Design, Setting, and Participants:**

Cross-sectional study of the Behavioral Risk Factor Surveillance System database, 2016 to 2017. The Behavioral Risk Factor Surveillance System is the largest national telephone-based survey of randomly sampled adults in the United States. A total of 892 394 participants with information on e-cigarette use and depression were included. Data analysis was conducted in May 2019.

**Exposures:**

Electronic cigarette use status defined by self-report as never, former, or current use.

**Main Outcomes and Measures:**

Self-reported history of a clinical diagnosis of depression.

**Results:**

Of the 892 394 participants (414 326 [29.0%] aged ≥60 years; 502 448 [51.3%] women), there were 28 736 (4.4%) current e-cigarette users, of whom 13 071 (62.1%) were aged between 18 and 39 years. Compared with never e-cigarette users, current e-cigarette users were more likely to be single, male, younger than 40 years, and current combustible cigarette smokers (single, 120 797 [24.3%] vs 10 517 [48.4%]; men, 318 970 [46.6%] vs 14 962 [60.1%]; aged 18-39 years, 129 085 [32.2%] vs 13 071 [62.1%]; current combustible cigarette use, 217 895 [7.9%] vs 8823 [51.8%]). In multivariable adjusted models, former e-cigarette users had 1.60-fold (95% CI, 1.54-1.67) higher odds of reporting a history of clinical diagnosis of depression than never users, whereas current e-cigarette users had 2.10 (95% CI, 1.98-2.23) times higher odds. Additionally, higher odds of reporting depression were observed with increased frequency of use among current e-cigarette users compared with never users (daily use: odds ratio, 2.39; 95% CI, 2.19-2.61; occasional use: odds ratio, 1.96; 95% CI, 1.82-2.10). Similar results were seen in subgroup analyses by sex, race/ethnicity, smoking status, and student status.

**Conclusions and Relevance:**

This study found a significant cross-sectional association between e-cigarette use and depression, which highlights the need for prospective studies analyzing the longitudinal risk of depression with e-cigarette use. If confirmed by other study designs, the potential mental health consequences may have regulatory implications for novel tobacco products.

## Key Points

**Question:**

What is the association between electronic cigarette (e-cigarette) use and depression?

**Findings:**

In this cross-sectional study of 892 394 participants in the Behavioral Risk Factor Surveillance System from 2016 to 2017, e-cigarette users had higher odds of reporting a history of clinical diagnosis of depression compared with participants who never used e-cigarettes. In addition, increased frequency of e-cigarette use was associated with incrementally higher odds of reporting depression.

**Meaning:**

These findings highlight the need for longitudinal studies to examine the association between e-cigarette use and depression, which may be bidirectional.

## Teaser

This cross-sectional study examines the association between electronic cigarette (e-cigarette) use and depression in a nationally representative sample of the adult population in the United States.

## Introduction

Electronic cigarettes (e-cigarettes) were introduced into the US market more than a decade ago as an alternative to combustible cigarettes.^1,2,3^ They are marketed as less harmful nicotine delivery devices; however, their contents are largely unregulated and vary widely, with nicotine concentrations ranging from 0 to 59 mg/mL.^4,5^ Many products also contain potentially toxic metals, such as arsenic and lead, as well as propylene glycol. When used, e-cigarettes may generate volatile organic compounds, including acetaldehyde and formaldehyde, especially at high temperatures.^4,6^

In 2016, 10.8 million adults (4.5%) in the United States reported current e-cigarette use, more than half of whom were younger than 35 years.^1^ Among current e-cigarette users, 3.6 million (33.5%) reported daily use, with 54.6% also reporting traditional cigarette smoking.^1^

Among individuals with mental health conditions (MHCs), who make up 36.4% of the US population,^7^ smoking rates are estimated to be 70% greater than in the general US population,^8^ with these individuals consuming approximately 56.4% of all cigarettes sold in the United States.^7^ Cigarette smokers with MHCs also tend to smoke more heavily and find it harder to quit smoking.^7,9^ The prevalence of depression in the population varies widely, from 6.7% to 16.6%,^10,11^ and a recent study found that prevalence of current e-cigarette use among those who reported having depression was 9.1%, compared with a prevalence of 4.5% in the general population.^1^

The popularity of e-cigarettes among the general population and their appeal to those who are susceptible to MHCs, including younger adults, raises a question regarding the association between e-cigarette use and mental health.^3,12^ To test our hypotheses that e-cigarette users might be more likely to have depression and that depressed individuals might be more likely to use e-cigarettes, we sought to examine the cross-sectional association between the use of e-cigarettes and depression in a large representative sample of adults in the United States.

## Methods

### Study Population

The Behavioral Risk Factor Surveillance System (BRFSS) is a nationwide, telephone-based survey of randomly sampled US residents older than 18 years, established by the US Centers for Disease Control and Prevention to collect information on health-related risk behaviors, chronic medical conditions, and the use of preventive health services.^13^ The survey includes participants in all 50 states as well as Washington, DC, and 3 US territories, making it the largest dynamic telephone-based health survey in the world.^13^

The BRFSS is a publicly available data set containing deidentified data. Thus, based on the code of federal regulations revised common rule,^14^ our analysis of the data set was exempt from institutional review board review. Each section of this report was prepared using the Strengthening the Reporting of Observational Studies in Epidemiology (STROBE) reporting guideline.

### Inclusion and Exclusion Criteria

To examine the association between e-cigarette use and depression, we pooled data from the 2016 and 2017 BRFSS surveys, which included 936 319 participants. Of the participants in the data set, we excluded participants with missing data on e-cigarette use (n = 39 729 [4.2%]), self-reported history of clinical diagnosis of depression (n = 3818 [0.4%]), and both (n = 378 [0.1%]), leaving a total sample size of 892 394 (95.3%).

### Use of e-Cigarettes

Participants were categorized as ever or never e-cigarette users based on their response to the question, “Have you ever used an e-cigarette or other electronic vaping product, even just 1 time, in your entire life?” Those who answered yes were then further classified into occasional or daily users based on their response to the question, “Do you now use e-cigarettes or other vaping products every day, some days, or not at all?” We classified e-cigarette smokers into 4 statuses, as follows: everyday e-cigarette user, some days e-cigarette user, former e-cigarette user, and non–e-cigarette user.

### Depression and Mental Health

The dependent variable of interest was depression, defined as a self-reported history of clinical diagnosis if participants answered yes to the question, “Has a physician ever told you have a depressive disorder (including depression, major depression, dysthymia, or minor depression)?”^15,16^ In separate supporting analyses, subjective poor mental health was explored as an alternative outcome, defined by evaluating participants’ response to the question, “Now thinking about your mental health, which includes stress, depression, and problems with emotions, for how many days during the past 30 days was your mental health not good?”^17^

### Covariate Assessment

Baseline demographic factors, including age, sex, race/ethnicity, marital status, education, and employment status, were self-reported according to BRFSS protocol. Income was defined using federal poverty line cutoffs for each state, taking into account the number of adults and children in the household for each state.^18^ Data on alcohol and combustible cigarette use were also collected. Heavy alcohol use was defined as more than 14 drinks per week for men and more than 7 drinks per week for women. Combustible cigarette smoking status was categorized as current, former, and never.

### Statistical Analysis

The BRFSS uses design weighting and iterative proportional fitting to ensure representativeness of the data.^19^ These weights were applied in all analyses done with the data set.

Sociodemographic and risk factor characteristics were described according to e-cigarette use categories by applying sampling design elements and using the svy subpop command. Multivariable logistic models were used to assess the association between e-cigarette use and depression, adjusting for age, sex, race/ethnicity, income, marital status, employment, education, heavy alcohol use, and combustible cigarette smoking. The prevalence of depression in the data set was high at 18.9%; thus, supplemental analyses were also performed using Poisson regression models to test the stability of the estimates. Given the known high prevalence of use of e-cigarettes among the younger population as well as among smokers, we performed subgroup analyses among college students and among combustible cigarette users.

Formal effect modification was tested using interaction terms for e-cigarette use and sex, age, race/ethnicity, and cigarette smoking. Stratified analyses were then performed and presented by these variables.

To further test our estimates, subjective mental health was explored as an alternative outcome. We compared those reporting 1 or more days of poor mental health with those reporting no days of poor mental health.

All analyses were conducted using Stata statistical software version 14.2 (StataCorp) with the significance level set at a 2-sided *P* < .05. Data analysis was conducted in May 2019.

## Results

Of 892 394 participants (414 326 [29.0%] aged ≥60 years; 502 448 [51.3%] women), 28 736 (4.4%) were current e-cigarette users, 111 337 (16.5%) were former e-cigarette users, and 752 321 (79.1%) were never e-cigarette users (Table 1). Compared with never e-cigarette users, current e-cigarette users were more likely to be single, men, and younger than 40 years and to concurrently use combustible cigarettes (single, 120 797 [24.3%] vs 10 517 [48.4%]; men, 318 970 [46.6%] vs 14 962 [60.1%]; aged 18-39 years, 129 085 [32.2%] vs 13 071 [62.1%]; current combustible cigarette use, 217 895 [7.9%] vs 8823 [51.8%]) (Table 1). Similar patterns were observed when comparing never e-cigarette users with former e-cigarette users (single, 120 797 [24.3%] vs 38 940 [46.7%]; men, 318 970 [46.6%] vs 55 727 [55.6%]; aged 18-39 years, 129 085 [32.2%] vs 48 808 [60.6%]; current combustible cigarette use, 217 895 [7.9%] vs 27 116 [46.7%]) (Table 1).

**Table 1.** Demographic and Socioeconomic Characteristics of Individuals in the BRFSS, 2016-2017, by e-Cigarette Use

Characteristic	No. (%)^a^		
	Current e-Cigarette User (n = 28 736)	Former e-Cigarette User (n = 111 337)	Never e-Cigarette User (n = 752 321)
Sex			
Men	14 962 (60.1)	55 727 (55.6)	318 970 (46.6)
Women	13 759 (39.9)	55 573 (44.4)	433 116 (53.4)
Age, y			
18-24	4817 (27.3)	15 361 (23.2)	29 687 (9.7)
25-29	2947 (13.4)	12 445 (14.5)	27 425 (6.5)
30-34	2769 (12.0)	11 226 (13.3)	33 531 (8.3)
35-39	2538 (9.4)	9776 (9.6)	38 442 (7.7)
40-44	2016 (8.1)	7946 (8.0)	39 998 (8.0)
45-49	2280 (6.6)	8554 (6.5)	49 351 (7.7)
50-54	2693 (7.4)	10 121 (7.5)	62 596 (9.4)
55-59	2919 (6.1)	11 255 (6.3)	76 665 (8.9)
≥60	5580 (9.6)	23 969 (11.0)	384 777 (33.8)
Race/ethnicity			
White	22 037 (71.9)	82 726 (66.2)	572 314(62.7)
Black or African American	1645 (8.6)	8100 (10.8)	60 516 (11.9)
Hispanic	1983(11.0)	9037 (15.0)	60 529 (17.2)
Other	2598 (8.5)	9632 (8.1)	46 013 (8.1)
Marital Status			
Married	10 000 (32.2)	41 450 (34.4)	413 777 (55.0)
Divorced	6329 (16.3)	23 386 (15.7)	110 882 (12.8)
Widowed	1725 (3.1)	6979 (3.2)	102 372 (7.9)
Single	10 517 (48.4)	38 940 (46.7)	120 797 (24.3)
Education			
<High school	2776 (14.3)	10 208 (13.8)	53 030 (13.4)
≥High school or some college	20 274 (72.9)	74 262 (68.9)	397 440 (56.4)
College graduate	5627 (12.8)	26 658 (17.4)	299 448 (30.2)
Employment status			
Employed	15 987 (61.9)	64 375 (63.1)	359 362 (55.3)
Unemployed	7815 (23.7)	26 521 (21.7)	117 920 (18.1)
Student	1533 (8.9)	5829 (8.9)	16 064 (5.1)
Retired	3192 (5.5)	13 856 (6.4)	253 949 (21.6)
Income, % of poverty line			
<100	4507 (17.6)	16 381 (17.4)	61 729 (13.3)
100-200	6864 (23.3)	26 066 (23.5)	142 894 (19.6)
>200	14 505 (59.1)	58 940 (59.2)	500 317 (67.1)
Heavy alcohol use	3183 (11.8)	12 047 (11.9)	36 081 (4.8)
Combustible cigarette smoking			
Never	3884 (19.5)	25 601 (30.5)	473 736 (67.9)
Current	8823 (51.8)	27 116 (46.7)	217 895 (7.9)
Former	15 887 (28.8)	58 085 (22.8)	56 486 (24.2)
Use of other tobacco products^b^	2062 (8.0)	7287 (6.6)	20 720 (2.8)
BMI			
<18.5	814 (3.1)	2423 (2.4)	10 692 (1.8)
18.5 to <25.0	9614 (36.5)	36 541 (36.5)	216 473 (31.9)
25.0 to <30.0	8998 (32.0)	35 665 (32.9)	256 803 (35.9)
≥30	8206 (28.4)	31 693 (28.2)	217 640 (30.4)
Any physical activity outside of work in last 30 d	19 947 (74.5)	77 806 (74.6)	548 009 (74.4)
Self-reported poor mental health days			
0	13 475 (47.0)	57 081 (50.4)	529 450 (68.9)
≥1	14 781 (53.0)	52 557(49.6)	211 535 (31.1)
History of clinical depression	10 633 (34.1)	34 197 (27.6)	124 032 (15.3)

Abbreviations: BMI, body mass index (calculated as weight in kilograms divided by height in meters squared); BRFSS, Behavioral Risk Factor Surveillance System; e-cigarette, electronic cigarette.

^a^ Percentages are weighted to represent the general population.

^b^ Defined as current use of chewing tobacco, snuff, or snus every day or some days.

**Figure 1.**
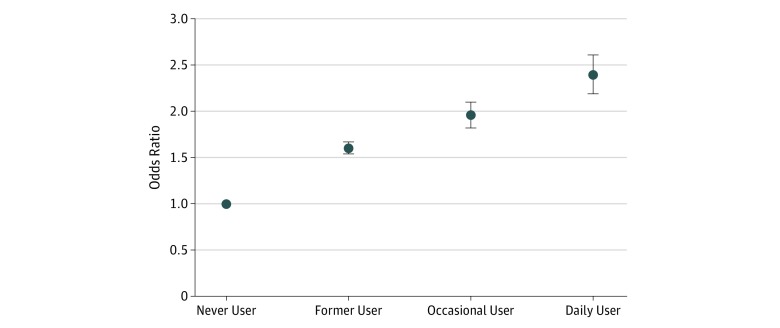
Association Between Electronic Cigarette Use and History of Clinical Diagnosis of Depression Odds ratios are adjusted for age, sex, race/ethnicity, income, marital status, education, employment, heavy alcohol use, and combustible cigarette use. Point estimates are represented with dots, and 95% CIs represented with upper and lower horizontal bars.

In multivariable-adjusted logistic regression analyses, compared with never users, former e-cigarette users had 1.60-fold (95% CI, 1.54-1.67) higher odds of reporting a history of clinical diagnosis of depression, while current e-cigarette users had 2.10-fold (95% CI, 1.98-2.23) higher odds. Among current e-cigarette users, we found graded higher odds of reporting depression among occasional and daily users compared with never users (occasional use: odds ratio [OR], 1.96; 95% CI, 1.82-2.10; daily use: OR, 2.39; 95% CI, 2.19-2.61) (Table 2 and Figure 1). Similar estimates were observed when multivariable-adjusted Poisson regression models were used to examine the association between e-cigarette use and depression (data not shown).

**Table 2.** Association Between e-Cigarette Use and History of Clinical Diagnosis of Depression and Subjective Poor Mental Health

e-Cigarette Use Status	OR (95% CI)			
	Clinical Diagnosis of Depression		Subjective Poor Mental Health	
	Unadjusted	Adjusted^a^	Unadjusted	Adjusted^a^
Never users	1 [Reference]	1 [Reference]	1 [Reference]	1 [Reference]
Former users	2.12 (2.06-2.18)	1.60 (1.54-1.67)	2.18 (2.13-2.24)	1.52 (1.47-1.57)
Current users	2.87 (2.74-3.01)	2.10 (1.98-2.23)	2.50 (2.39-2.62)	1.67 (1.58-1.76)
Occasional use	2.73 (2.58-2.89)	1.96 (1.82-2.10)	2.64 (2.50-2.80)	1.73 (1.61-1.85)
Daily use	3.17 (2.94-3.42)	2.39 (2.19-2.61)	2.25 (2.09-2.42)	1.57 (1.44-1.70)

Abbreviations: e-cigarette, electronic cigarette; OR, odds ratio.

^a^ Adjusted for age, sex, race/ethnicity, income, marital status, education, employment, heavy alcohol use, and combustible cigarette use.

**Figure 2.**
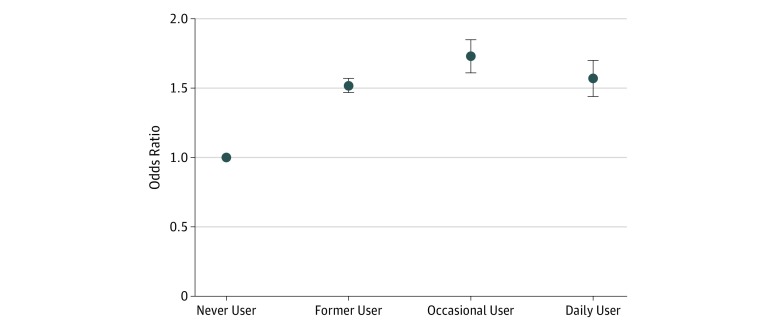
Association Between Electronic Cigarette Use and Subjective Poor Mental Health Odds ratios are adjusted for age, sex, race/ethnicity, income, marital status, education, employment, heavy alcohol use, and combustible cigarette use. Point estimates are represented with dots, and 95% CIs represented with upper and lower horizontal bars.

In separate supporting analyses using self-reported days of poor mental health as a binary dependent variable, compared with never users who reported no days of poor mental health, former users had 1.52-fold (95% CI, 1.47-1.57) higher odds of reporting at least 1 day of poor mental health, while current users had 1.67-fold (95% CI, 1.58-1.76) higher odds. Similarly, occasional and daily users were more likely to report at least 1 day of poor mental health compared with never users (occasional use: OR, 1.73; 95% CI, 1.61-1.85; daily use: OR, 1.57; 95% CI 1.44-1.70) (Table 2 and Figure 2).

Among never cigarette smokers, current e-cigarette users were 2.16-fold (95% CI, 1.87-2.49) more likely to report depression compared with never e-cigarette users. These results were similar when comparing dual users to current smokers who never used e-cigarettes (OR, 2.11; 95% CI, 1.94-2.30) (Table 3).

**Table 3.** Association Between e-Cigarette Use and Depression by Combustible Cigarette Smoking Status

e-Cigarette Use Status	OR (95% CI)^a^		
	Never Smoker (N = 503 221)	Former Smoker (N = 253 834)	Current Smoker (N = 130 458)
Never user	1 [Reference]	1 [Reference]	1 [Reference]
Former user	1.63 (1.52-1.76)	1.59 (1.48-1.71)	1.57 (1.48-1.66)
Current user	2.16 (1.87-2.49)	1.89 (1.71-2.10)	2.11 (1.94-2.30)

Abbreviations: e-cigarette, electronic cigarette; OR, odds ratio.

^a^ Adjusted for age, sex, race/ethnicity, income, marital status, education, employment, heavy alcohol use.

Both men and women who were current e-cigarette users were twice as likely to report depression compared with never e-cigarette users (men: OR, 2.17; 95% CI 2.00-2.35; women: OR, 2.05; 95% CI, 1.88-2.24) (eTable 1 in the Supplement). Similar results were also observed across strata of age and race, and among students (eg, current users aged 18-24 years: OR, 2.22; 95% CI, 1.92-2.57; current users who are Hispanic: OR, 2.45; 95% CI, 1.99-3.03; current users who are students: OR, 2.01; 95% CI, 1.58-2.56) (eTable 2, eTable 3, and eTable 4 in the Supplement).

In subgroup analyses, we observed statistically significant interactions between e-cigarette use and age (OR per increase in age category, 0.97; 95% CI, 0.96-0.97; *P* < .001), sex (women vs men: OR, 1.13; 95% CI, 1.08-1.18; *P* < .001), and race/ethnicity (white individuals vs individuals of other races/ethnicities: OR, 1.07; 95% CI, 1.04-109; *P* < .001). However, point estimates of the association between e-cigarette use and depression were qualitatively consistent across all subgroups, with a consistently increased odds of depression with former e-cigarette use and an even stronger association with current e-cigarette use. No statistically significant interactions were observed between e-cigarette use and cigarette smoking (OR, 1.03; 95% CI, 1.00-1.10; *P* = .09).

## Discussion

In this cross-sectional analysis of a large nationally representative survey of adults in the United States, we found that former and current e-cigarette users were more likely to report a history of clinical diagnosis of depression compared with never users. In addition, we found a graded association between frequency of use and depression. We further explored subgroups defined by age and race and found no significant differences in the association between e-cigarettes and depression or mental health, suggesting that the association between e-cigarettes and these mental health outcomes was similar across these broad segments of the population.

Our study provides additional evidence to establish an association between e-cigarette use and depression, which could have potentially significant implications for public health, clinical practice, and health policy. Our results are supported by findings from a small longitudinal study by Lechner et al^20^ conducted among adolescents in Los Angeles, California, which showed that sustained e-cigarette use was associated with an increase in depression symptoms over a 12-month period. While cross-sectional, our sample size was larger, enabling us to conduct important subgroup analyses.

Similar to a study that was conducted in France in 2019,^21^ we also found that the association between e-cigarettes and depression did not differ significantly by sex or among current and former cigarette smokers. However, while that study found no association between e-cigarettes and depression in nonsmokers, we found that never cigarette smokers who used e-cigarettes had twice the odds of reporting a clinical diagnosis depression compared with those who did not use e-cigarettes, an estimate similar to that found among dual users.

Previous work has shown that college students are more likely to explore new products, including e-cigarettes, and it is not uncommon for this group to be targeted by tobacco companies’ marketing strategies.^22,23^ Therefore, we explored the association between e-cigarette use and depression in the subpopulation and found that current e-cigarette users also had twice the odds of reporting depression compared with never users in the same category. This highlights the potential susceptibility of e-cigarette users in this group to depression.

Prolonged nicotine exposure has been shown to disrupt the cerebral dopamine pathway, amplify stress sensitivity, and distort the coping mechanisms that buffer against depressive symptoms.^20^ As many e-cigarettes contain nicotine,^4,24^ this could explain the graded association seen with increased frequency of use and depression, suggesting a potential dose-response relationship. In addition to nicotine, e-cigarettes contain varying amounts of trace metals including arsenic, aluminum, and lead,^25,26,27^ many of which are on the priority pollutant list of the Agency for Toxic Substances and Disease registry, with known adverse health effects.^26^ In particular, lead and aluminum affect the central and peripheral nervous systems and may potentially contribute to the observed association between e-cigarettes and depression.^27^

Traditional cigarette smoking affects the metabolism of a number of psychiatric medications, mainly via the induction of metabolic enzymes, leading to reduced blood levels and reduced therapeutic effectiveness.^28,29^ Analyses of e-cigarette contents have shown that they also contain tobacco-specific nitrosamines and some volatile organic compounds, although in less quantity than observed in traditional cigarettes.^30,31^ This raises further concern about the use of e-cigarettes among individuals with MHCs, as their use might complicate the treatment of psychiatric conditions.

Furthermore, a higher frequency of regular cigarette smoking has been reported among people with major depression compared with individuals who have never had major depression or any psychiatric illness,^32,33^ and a brief prospective study done in adolescents found smoking to increase the risk of developing an episode of major depressive disorder.^34^ Among traditional cigarette smokers, patients with depression are less likely to successfully quit smoking, possibly because of worsening depressive symptoms during quitting attempts and poor tolerance of nicotine withdrawal symptoms.^21^ Smoking has also been shown to be highly predictive of future suicidal behavior, making it particularly concerning in individuals with a history of depression.^35^ Considering the similarities between e-cigarettes and traditional cigarettes, individuals with depression might be more susceptible to sustained use of e-cigarettes and may find it harder to reduce or quit their e-cigarette use. Furthermore, e-cigarette use might predispose users to developing major depression in the long term.

Consistent evidence of the gateway theory of an association between e-cigarette use and subsequent cigarette smoking initiation has been reported in a meta-analysis done by Soneji et al.^36,37,38^ This theory, as well as the well-established addictive nature of nicotine,^39,40^ make the rapid uptake of e-cigarettes among teenagers—described as an epidemic by the US Food and Drug Administration^41^ and the US Surgeon General^42^—particularly concerning, especially because our findings show no difference in association between e-cigarette use and depression among younger and older adults.

Our findings, if confirmed in other study designs with longitudinal follow-up, may provide data to inform policies that could protect populations susceptible to depression. For example, the association between e-cigarette use and depression might justify further regulation of advertisements and marketing strategies, appropriate warning labels that highlight the potential risk of depression associated with e-cigarette use, and public health education about the potential effects of e-cigarettes, especially among those with MHCs. This association could also inform clinical practice by providing information that physicians could consider in counselling patients seeking information about the use of e-cigarettes, especially those with depression. At the very least, our findings warrant careful and thorough evaluation of e-cigarette use in both youth and adults with depression. Physicians should consider routine collection of information pertaining to e-cigarette use during clinic visits, especially in patients with depression, and routine counseling for those who use e-cigarettes, offering support to those who express willingness to quit.

### Strengths and Limitations

To our knowledge, this study was the first to examine the association between e-cigarette use and depression across several subpopulations in the largest nationally representative database in the United States. However, our study has limitations. It was observational and cross-sectional, and therefore, we can neither infer causality nor ascertain the direction of the association, which we submit might be bidirectional. Furthermore, we lacked granular information on product characteristics, such as the brands of e-cigarettes used, and detailed exposure characterization, including frequency or duration of vaping. We also cannot exclude the possibility of residual confounding. Finally, data on both the exposure and outcome were self-reported, which might lead to nonrandom misclassification.

## Conclusions

Our results suggest a strong association between e-cigarette use and depression, and this may have potential implications for regulation of e-cigarettes. Our results also highlighted the need for longitudinal studies to investigate the risk of depression associated with e-cigarette use and the potential bidirectionality of the association between e-cigarette use and depression.

## Notes

Supplement. **eTable 1. **Association Between e-Cigarette Use and Depression, Stratified by Sex **eTable 2. **Association Between e-Cigarette Use and Depression, Stratified by Age **eTable 3. **Association Between e-Cigarette Use and Depression, Stratified by Race/Ethnicity **eTable 4. **Association Between e-Cigarette Use and Depression Among Students
