# Supplementary material for: Association Between e-Cigarette Use and Depression in the Behavioral Risk Factor Surveillance System, 2016-2017
Source: JAMA Netw Open. 2019 Dec 4;2(12):e1916800. doi: 10.1001/jamanetworkopen.2019.16800 (PMC6902792; doi:10.1001/jamanetworkopen.2019.16800)
Supplement: Supplement. — eTable 1. Association Between e-Cigarette Use and Depression, Stratified by Sex eTable 2. Association Between e-Cigarette Use and Depression, Stratified by Age eTable 3. Association Between e-Cigarette Use and Depression, Stratified by Race/Ethnicity eTable 4. Association Between e-Cigarette Use and Depression Among Students [file jamanetwopen-2-e1916800-s001.pdf]

Supplementary Online Content

Obisesan OH, Mirbolouk M, Osei AD, et al. Association between e-cigarette use and depression in the Behavioral Risk Factor Surveillance System, 2016-2017. *JAMA Netw Open*. 2019;2(12):e1916800. doi:10.1001/jamanetworkopen.2019.16800

eTable 1. Association Between e-Cigarette Use and Depression, Stratified by Sex
eTable 2. Association Between e-Cigarette Use and Depression, Stratified by Age
eTable 3. Association Between e-Cigarette Use and Depression, Stratified by Race/Ethnicity
eTable 4. Association Between e-Cigarette Use and Depression Among Students

This supplementary material has been provided by the authors to give readers additional information about their work.

© 2019 Obisesan OH et al. *JAMA Network Open.*

eTable 1. Association Between e-Cigarette Use and Depression, Stratified by Sex

E-cigarette use status	Women<br>OR (95%CI)<br>(N=502,448)	Men<br>OR (95%CI)<br>(N=389,659)
**Never users**	**Ref**	**Ref**
**Former Users**	1.71 (1.62, 1.80)	1.48 (1.39, 1.56)
**Current Users**	2.17 (2.00,2.35)	2.05 (1.88, 2.24)

*Adjusted for age, sex, race, income, marital status, education, employment, cigarette smoking, heavy alcohol use.

© 2019 Obisesan OH et al. *JAMA Network Open.*

eTable 2. Association Between e-Cigarette Use and Depression, Stratified by Age

E-cigarette use status	18-24 years (N=49,865) OR (95% CI)	25-29 years (N=42,817) OR (95% CI)	30-34 years (N=47,526) OR (95% CI)	35-39 years (N=50,756) OR (95% CI)	40-44 years (N=49,960) OR (95% CI)	45-49 years (N=60,185) OR (95% CI)	≥ 50years (N=580,575) OR (95% CI)
**Never users**	**Ref**	**Ref**	**Ref**	**Ref**	**Ref**	**Ref**	**Ref**
**Former users**	1.64 (1.47,1.83)	1.70 (1.51,1.91)	1.63 (1.45,1.84)	1.57 (1.38,1.77)	1.54 (1.34,1.77)	1.47 (1.29,1.68)	1.65 (1.55,1.75)
**Current users**	2.22 (1.92,2.57)	2.13 (1.80,2.52)	2.11 (1.77,2.52)	2.26 (1.87,2.73)	1.70 (1.37, 2.11)	1.92 (1.54,2.41)	2.06 (1.88,2.26)

*Adjusted for age, sex, race, income, marital status, education, employment, cigarette smoking, heavy alcohol use.

© 2019 Obisesan OH et al. *JAMA Network Open.*

eTable 3. Association Between e-Cigarette Use and Depression, Stratified by Race/Ethnicity

E-cigarette use status	White OR (95%CI) (N=677,077)	Black OR (95%CI) (N=70,261)	Hispanic OR (95%CI) (N=71,549)	Other OR (95%CI) (N=58,243)
**Never users**	**Ref**	**Ref**	**Ref**	**Ref**
**Former Users**	1.52 (1.46,1.59)	1.39 (1.22, 1.58)	1.91 (1.67, 2.17)	2.02 (1.72, 2.37)
**Current Users**	2.01 (1.88, 2.14)	1.96 (1.56, 2.48)	2.45 (1.99, 3.03)	2.38 (1.86, 3.04)

*Adjusted for age, sex, race, income, marital status, education, employment, cigarette smoking, heavy alcohol use.

© 2019 Obisesan OH et al. *JAMA Network Open.*

eTable 4. Association Between e-Cigarette Use and Depression Among Students

E-cigarette use status	Students OR (95%CI) (N=23,426)
**Never users**	**Ref**
**Former Users**	1.57 (1.33,1.86)
**Current Users**	2.01 (1.58,2.56)

*Adjusted for age, sex, race, income, marital status, education, employment, cigarette smoking, heavy alcohol use.

© 2019 Obisesan OH et al. *JAMA Network Open.*
